# The bud dormancy disconnect: latent buds of grapevine are dormant during summer despite a high metabolic rate

**DOI:** 10.1093/jxb/erac001

**Published:** 2022-01-11

**Authors:** Yazhini Velappan, Tinashe G Chabikwa, John A Considine, Patricia Agudelo-Romero, Christine H Foyer, Santiago Signorelli, Michael J Considine

**Affiliations:** 1 ARC Centre of Excellence in Plant Energy Biology, and the School of Molecular Sciences, University of Western Australia, Perth, WA 6009, Australia; 2 The UWA Institute of Agriculture, University of Western Australia, Perth, WA 6009, Australia; 3 School of Biosciences, University of Birmingham, Edgbaston B15 2TT, UK; 4 Departamento de Biología Vegetal, Universidad de la República, Montevideo, 12900, Uruguay; 5 Department of Primary Industries and Regional Development, South Perth, WA 6151, Australia; 6 CNB-CSIC, Spain

**Keywords:** Acclimation, bud dormancy, chilling, perennial plant, phenology, respiration, seasonality temperature response, transcription

## Abstract

Grapevine (*Vitis vinifera* L.) displays wide plasticity to climate; however, the physiology of dormancy along a seasonal continuum is poorly understood. Here we investigated the apparent disconnect between dormancy and the underlying respiratory physiology and transcriptome of grapevine buds, from bud set in summer to bud burst in spring. The establishment of dormancy in summer was pronounced and reproducible; however, this was coupled with little or no change in physiology, indicated by respiration, hydration, and tissue oxygen tension. The release of dormancy was biphasic; the depth of dormancy declined substantially by mid-autumn, while the subsequent decline towards spring was moderate. Observed changes in physiology failed to explain the first phase of dormancy decline, in particular. Transcriptome data contrasting development from summer through to spring also indicated that dormancy was poorly reflected by metabolic quiescence during summer and autumn. Gene Ontology and enrichment data revealed the prevailing influence of abscisic acid (ABA)-related gene expression during the transition from summer to autumn, and promoter motif analysis suggested that photoperiod may play an important role in regulating ABA functions during the establishment of dormancy. Transcriptomic data from later transitions reinforced the importance of oxidation and hypoxia as physiological cues to regulate the maintenance of quiescence and resumption of growth. Collectively these data reveal a novel disconnect between growth and metabolic quiescence in grapevine following bud set, which requires further experimentation to explain the phenology and dormancy relationships.

## Teaser

During summer, latent buds of grapevine are metabolically and transcriptionally active despite the inability to resume growth, which coincides with enrichment of photoperiod and ABA signalling processes.

## Introduction

Dormancy is a seasonally entrained condition of quiescence that is common among many woody perennial plant species. The transition to dormancy serves to suspend vegetative and reproductive growth and protect embryonic organs during conditions where seasonal cues are unfavourable (Singh *et al.*, 2017; Friedman, 2020). Understanding the ecophysiology of dormancy transitions is important for managing the effects of local and global climate change in forest and crop systems. Grapevine (*Vitis vinifera* L.) is the most economically important fruit crop worldwide, grown commercially on all continents except Antarctica (http://faostat.fao.org/). Contemporary knowledge of physiology and molecular regulation of dormancy in grapevine remains limited to the exit of dormancy and transition to bud burst (Keilin *et al.*, 2007; Ophir *et al.*, 2009; Vergara *et al.*, 2012; Rubio *et al.*, 2014; Meitha *et al.*, 2015, 2018; Sudawan *et al.*, 2016; Pérez and Noriega, 2018; Shi *et al.*, 2018; Signorelli *et al.*, 2018; Zheng *et al.*, 2018*a*, *b*). In particular, knowledge of the relationship between growth quiescence (syn. dormancy) and metabolic or cellular quiescence during the early stages of dormancy, following bud set in late summer is limited.

The axillary bud is quiescent during development and ramification, largely suppressed from outgrowth by correlative inhibition by the main shoot apex (N) and prompt bud (N+1, summer lateral) (Lavee and May, 1997). Photoperiod (Fennell and Hoover, 1991; Wake and Fennell, 2000) and physical maturation processes (Pouget, 1963) influence the subsequent transition into dormancy *sensu stricto*; however, the transition is diffuse (Lavee and May, 1997). Data of several grapevine varieties from 26° to 34° latitude suggest that the depth of dormancy increases to a maximum prior to, or during, early winter (Lavee and May, 1997; Rubio *et al.*, 2016; Parada *et al.*, 2016; Zheng *et al.*, 2018*a*). Several studies show that cold hardiness and responses to chilling correlate well with bud phenology and bud burst in autumn and winter (Ferguson *et al.*, 2011, 2014; Kovaleski *et al.*, 2018; Kovaleski and Londo, 2019). However, a selection of studies illustrate a distinct behaviour, where the depth of dormancy shows a pronounced peak in late summer before declining prior to winter (Pouget, 1963; Nigond, 1967; Cragin, 2015). These disparities indicate that the contemporary view of dormancy release as a solely temperature-driven phenomenon may need to be reconsidered (Jewaria *et al.*, 2021).

To date, the most extensive temporal, developmental dataset of grapevine buds revealed pronounced circannual rhythms in gene expression of homologues of flowering pathway regulators, as well as major functional categories of genes, such as photosynthesis and regulation of the cell cycle (Diaz-Riquelme *et al.*, 2012). No physiological data were presented, however, which limits context and application to the understanding of the metabolic and cellular regulation underpinning dormancy.

This study sought to establish a physiology and transcriptome platform to investigate the climate and seasonal dependence of bud dormancy in cultivated grapevine. The data reveal a pronounced peak of dormancy in late summer followed by a biphasic release from dormancy during autumn and late winter. Dormancy *sensu stricto* was substantially uncoupled from changes in primary metabolism or contemporary models of chilling units. Gene Ontology (GO) and enrichment of RNA sequencing (RNAseq) data provided evidence that photoperiod- and abscisic acid (ABA)-dependent processes may govern early season (summer) changes in transcription. These data provide new insight into the onset of dormancy in grapevine and particularly the relationship to seasonality and environmental cues such as light and temperature.

## Materials and methods

Unless otherwise stated, all chemicals were supplied by Sigma Aldrich, NSW, Australia.

### Plant material

Material for this study was collected from 275 similarly vigorous, non-consecutive vines of *V. vinifera* (L.) cv. Merlot (clone FVD3v14/VX/UCD on own roots), across six rows in a commercial vineyard from the Margaret River region in Western Australia, Australia (33°47ʹS, 115°02ʹE). The Margaret River region has a maritime temperate climate, with mean annual temperatures of 10.7–21.4 °C, predominantly winter rainfall of 957 mm per annum, and elevation 80 m (http://www.bom.gov.au/climate/averages/tables/cw_009746.shtml). Merlot is typically pruned in the first week of July, with bud burst occurring in early September. The vines used in this study were not pruned until following the final sampling in September, at which point <10% of buds had burst in the field. Vines used in this study were not treated with hydrogen cyanamide (H_2_CN_2_) in the field.

**Fig. 1.**
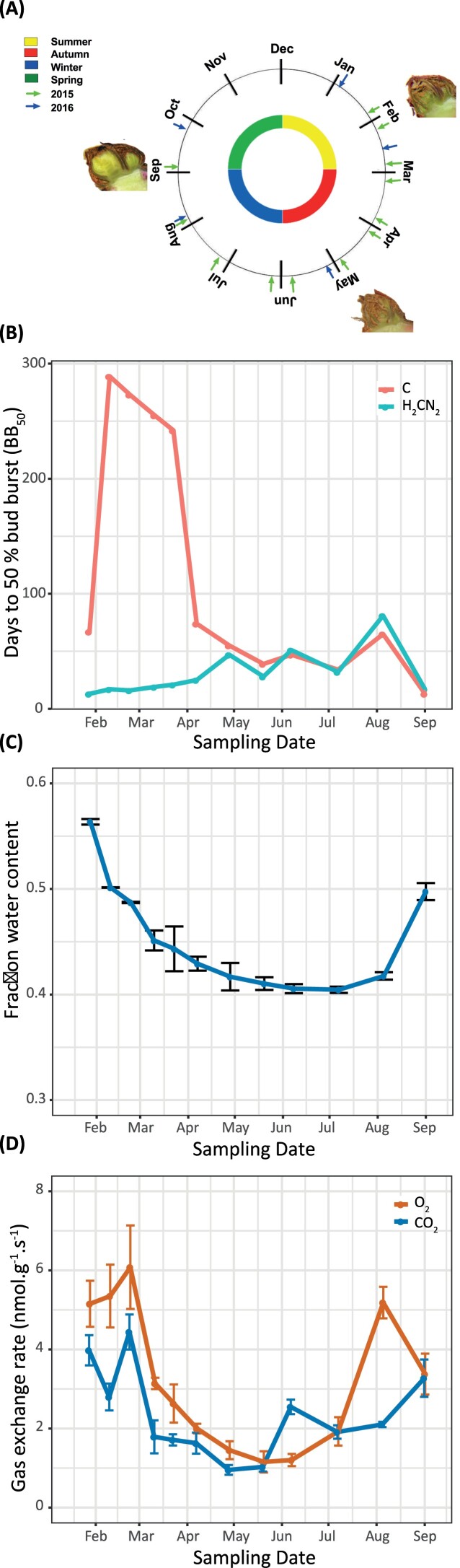
Dormancy and physiology of grapevine buds sampled throughout the season in the Margaret River region of Western Australia (2015, 33°S latitude). (A) Phenological calendar showing the sampling dates for 2015 and 2016 in this study and corresponding seasons. The calendar position of the bud images indicates the sample times used for RNA sequencing. (B) The depth of dormancy of single-node explants collected in 2015 and grown in forcing conditions, expressed as the time to 50% bud burst (BB_50_; *n*=50). Control (C) and buds treated with 0.31 M hydrogen cyanamide (H_2_CN_2_). (C) Water content (*n*=3) and (D) respiration (*n*=4) of samples collected at the corresponding dates. Vertical bars on (C) and (D) represent *s*_x_. Data for 2016 are provided in Supplementary Table S2.

Prior to the study, the canes were tagged from numbered vines and randomly assigned to collection dates. Canes of diameter 5–12 mm, comprising nodes 2–11 acropetally (where node 1 is the node above the first internode >7 mm) were collected from specific vines from mid-summer in January through to spring in September in the years 2015 and 2016 on 12 sampling dates (28 January, 10 February, 23 February, 11 March, 23 March, 7 April, 28 April, 20 May, 7 June, 7 July, 5 August, and 1 September) in 2015 and five sampling dates (6 January, 15 February, 10 May, 10 August, and 23 September) in 2016 (Fig. 1A). At the earliest sampling time (January), all basal buds up to node >15 were mature and lignified (data not shown). Immediately following sampling, the canes were stored at 20 °C in the dark for <48 h, during which time all respiratory and tissue oxygen partial pressure (pO_2_) analyses were performed. Nodes were randomly assorted to the assays, such that a single node is the basic biological unit, and, where necessary, multiple buds were pooled into one biological replicate, as described for each method herein. Buds for the gene expression profiling (RNAseq) were snap-frozen at sampling and stored at –80 °C. All the material was collected between 07.00 h and 10.00 h, to avoid changes in gene expression due to circadian regulation.

### **Depth of dormancy (BB**_**50**_)

Bud dormancy was measured using single node explants, which is a commonly used system to study the physiology of early shoot and inflorescence development in grapevine (Pouget, 1963; Mullins, 1966; Nigond, 1967; Antolín *et al.*, 2010), and excludes the influence of adjacent or distant organs. Each explant comprised ~50 mm of cane beneath and 10 mm cane above the node, diameter ~7–12 mm. Explants were grown in potting mix [pH ~6.0 fine composted pine bark:coco peat:brown river sand, ratio 2.5:1:1.5 (w/w/w)] in a controlled-temperature room at 20 °C, 12 h photoperiod, illuminated with fluorescent light at 100 µmol m^−2^ s^−1^. Soil moisture was maintained at >80% of water-holding capacity.

Because bud burst varies stochastically (Lavee and May, 1997), 50 buds were used to represent the dormancy state of the population at the time of sampling. H_2_CN_2_ was used as a positive control, as it is a widely used dormancy-breaking agent and has also been widely studied in the context of bud dormancy (Or *et al.*, 2000; Ophir *et al.*, 2009).

For the H_2_CN_2_ treatment, 50 buds were immersed in 2.5% (v/v) Dormex® (Crop Care, Australasia; equal to 0.31 M H_2_CN_2_) for 20 s and air dried prior to transplanting. The untreated buds were immersed in distilled water for 20 s and air dried prior to planting. The stage of bud burst was scored at the emergence of visible green leaf tips (EL4), according to the modified Eichorn–Lorenz scale (Coombe, 1995). Bud burst was recorded three times per week for up to 350 d or until 100% bud burst, and the depth of dormancy was calculated as the time to reach 50% bud burst (25/50 buds burst; BB_50_).

### Climate and chilling data

The weather data were obtained from the Moss Wood vineyard in Margaret River, Western Australia for 2015 and 2016. Average daily mean air temperatures were calculated from hourly air temperatures using the R statistical package (R Core Team, 2020) and plotted using the ggplot2 package of R (Wickham, 2009) as scatter dot plots fitted with a quadratic spline with degree of freedom (df)=4 and degree=2. The photoperiod data were obtained from the website https://www.usno.navy.mil/USNO/astronomical-applications/data-services/ and plotted using the ggplot2 package of R (Wickham, 2009) as a line plot.

Chilling data were modelled by three methods; the Daily Positive Utah Chill Unit (DPCU) model (Linsley-Noakes and Allan, 1994), the Utah model (Richardson *et al.*, 1974), and the base 7.2 °C model. The cumulative chilling was calculated for each day from the raw data and represented as a line plot using the R package (R Core Team, 2020) and the ggplot2 package of R (Wickham, 2009), respectively.

### Bud moisture content

Ten buds per biological replicate (three biological replicates) were transversely sectioned from the canes and their fresh weight was recorded. All buds were inspected for signs of necrosis prior to including them in the analysis. Dry weight was calculated post-drying at 60 °C for 7 d, and moisture content was calculated as g H_2_O 100 g^–1^ FW.

### Bud respiration

Five buds per biological replicate (three biological replicates) were transversely sectioned from the canes, weighed immediately, and placed on a thin agar plate, sectioned side down, to prevent dehydration and gas exchange from the cut base. Rate of O_2_ uptake and CO_2_ release were calculated.

#### O_2_ uptake

The rate of O_2_ uptake for every biological replicate was measured in the dark using the Unisense MicroRespiration system (OX-MR, Unisense, Denmark) with a Clark-type O_2_ microsensor in a 4 ml respiration chamber at a constant temperature of 20 °C (Shaw *et al.*, 2017). The readings were obtained using the SensorTrace RATE software (Unisense, Denmark).

#### CO_2_ release

The rate of CO_2_ release was measured in the dark, in an insect respiration chamber (6400-89; Li-COR, Lincoln, NE, USA) attached to a Li-6400XT portable gas exchange system at 20 °C, in CO_2_-controlled air (380 µmol CO_2_ mol^–1^ air) with 100 µmol m^–1^ s^–1^ air flow, at 55–75% relative humidity. The measurements were recorded once the ‘stableF’ value was read 1 (i.e. after stabilization of humidity, CO_2_, and air flow) following transfer of sample to the chamber. The readings obtained were later analysed.

### **Internal bud O**_**2**_ **partial pressure (pO**_**2**_)

The internal pO_2_ of 3–4 biological replicates in 2015 (six in 2016) with one single bud cutting per replicate was measured in the dark at 20 °C using a Clark-type oxygen micro-sensor with a tip diameter of 25 µm (OX-25; Unisense A/S, Aarhus, Denmark). The microelectrode was calibrated at atmospheric pO_2_ of 20.87 kPa and at zero pO_2_ (100% nitrogen gas) and then mechanically guided into the bud, starting from the outer scale surface at 0 µm to the inner meristematic core at 2000 µm, in 25 µm steps with a stabilizing pause of 3 s in between steps with the aid of a motorized micro-manipulator, as previously described (Shaw *et al.*, 2017). The values were recorded automatically at the end of each step (i.e. every 25 µm). The readings were processed using the SensorTrace RATE software (Unisense, Denmark), analysed using the R statistical package (R Core Team, 2020), and presented in graphical form using the ggplot2 package of R (Wickham, 2009), with data fitted with a LOESS regression curve at 95% confidence intervals (*n*=3–6 per month per year).

### Physiological data analysis and statistics

All calculations were performed using Microsoft Excel 2016 and the R statistical package (R Core Team, 2020), and graphics were compiled using the ggplot2 package of R (Wickham, 2009). At least three biological replicates were used per analysis. Significant differences among various sampling dates were corroborated statistically by applying one-way ANOVA test, using Tukey’s honestly significant difference (HSD) post-hoc test with *P*≤0.01.

### RNA isolation, library preparation, and RNAseq

RNA extractions and libraries (three biological replicates of 2–3 buds each per condition, ~50 ng) were prepared from material collected on the corresponding dates (Fig. 1A) and prepared for sequencing as described in Meitha *et al.* (2018), with minor modifications. Libraries were prepared with the TruSeq Stranded Total RNA with the Ribo-Zero Plant Kit (Illumina, Scorseby, Australia) according to the manufacturer’s instructions. RNAseq was performed on an Illumina HiSeq2500 by Novogene (Hong Kong) at ~9 Gb of data of 150 pair-end (PE) reads per library. Raw data files have been submitted to NCBI (BioProject ID PRJNA575976, http://www.ncbi.nlm.nih.gov/bioproject/575796).

### RNAseq data processing and analysis

Transcriptomic data analysis was performed according to Meitha *et al.* (2018) with minor modifications, and summary statistics of read length and read mapping are provided in Supplementary Table S1. Briefly, FastQC software was used to assess the quality of the fastq files (https://www.bioinformatics.babraham.ac.uk/projects/fastqc/). One of the libraries (Sample S1_1) did not meet quality control and was excluded from the analysis. Adapter and quality trimming were performed using Trimmomatic (Bolger *et al.*, 2014) with default settings. Salmon (Patro *et al.*, 2017), a pseudoalignment algorithm, was used to map the trimmed reads to the 12X v2.1 *V. vinifera* PN40024 reference genome (Canaguier *et al.*, 2017) with sequence-specific and GC bias correction, and quantify gene expression. The grapevine reference genome and annotation were obtained from the Phytozome v.12.1 database (Goodstein *et al.*, 2012). The raw counts matrix obtained from Salmon was read into edgeR (Robinson *et al.*, 2010), then normalized using the trimmed mean of M values (TMM) method to log counts per million reads (logCPM) and filtered to remove genes expressed at a low level (i.e. genes with <1 read in any sample). Graphical representations of the data before and after normalization are presented in Supplementary Fig. S1A, B. The quality of the replicates was checked using unsupervised clustering of samples before and after normalization and are presented in the multidimensional scaling (MDS) plot (Supplementary Fig. S1C). The normalized counts for each gene were further transformed and fitted into a linear model using the Voom function from limma (Law *et al.*, 2014). Mean variance relationships were evaluated before and after Voom precision weights were applied to the data (Supplementary Fig. S2).

Differentially expressed gene (DEG) analysis was carried out using edgeR and limma (Robinson *et al.*, 2010; Ritchie *et al.*, 2015) Bioconductor packages with default settings. *P*-values were corrected for multiple testing using the Benjamini–Hochberg method [false discovery rate (FDR) ≤0.01] (Benjamini and Hochberg, 1995). The data were then filtered to consider only genes with an absolute log_2_fold change ≥1 (log_2_FC |1|).

### Functional enrichment analysis

Three lists of DEGs from each comparison, up- and down-regulated genes and all (up- plus down-regulated genes) were used to perform a functional enrichment analysis using GOstats (Falcon and Gentleman, 2007) in R. This was done to identify significant functional categories of the genes based on *V. vinifera* functional classification of 12X v. 2.1. GOstats uses the hypergeometric distribution (a widely used method to test for over-representation) to compare each DEG list with the list of total genes. GO terms with *P*<0.05 were considered to be significantly enriched. All plots were rendered using the gplots and ggplots programs in R.

### Known *cis*-regulatory motif analysis

Grapevine promoter sequences (1.2 kb upstream of the coding sequence) of all annotated *V. vinifera* 12X v.2.1 genes (which is referred to as the background) were obtained from the Grape Genome Database (Vitulo *et al.*, 2014). Subsets of promoter sequences of DEGs from the three comparisons February–April, April–June, and June–September were retrieved from all grapevine gene promoter sequences (i.e. the background). Each gene set was analysed for enriched transcription factor-binding motifs/*cis*-elements using by using Homer v.4.11 (Heinz *et al.*, 2010) findMotifs.pl script with default parameters.

## Results

### The within-year dynamics of dormancy and physiology of grapevine buds

We evaluated the competence of bud growth of cv. Merlot explants sampled from mid-summer through to early spring over two consecutive years (Margaret River, Australia; 33°47ʹS, 115°02ʹE). The first sample point was in late January (~4 weeks after the summer solstice on 22 December). The data revealed a pronounced but transient peak in bud dormancy (BB_50_) during the period from late January to April, reaching a climax during February/March (Fig. 1B). The BB_50_ declined at a similar rate to that at which it developed, from late March to early April. Thereafter the BB_50_ continued to decline at a more linear but steady rate towards August/September, at the beginning of spring (Fig. 1B). Between 70% and 80% of buds sampled during the summer/autumn climax period burst within 350 d and those remaining were necrotic. Positive control buds treated with H_2_CN_2_ reached >95% bud burst (Fig. 1B, see below), indicating that the necrotic buds were viable at the time of sampling. Notwithstanding, the buds sampled during the February/March period were remarkably resilient. These trends were consistent in both 2015 and 2016 (Supplementary Table S2), and consistent with the patterns observed in cv. Merlot and cv. Carignan in France (Pouget, 1963; Nigond, 1967).

The application of H_2_CN_2_ dramatically accelerated the rate of bud burst (BB_50_) when applied to explants sampled during the period from February through to April (Fig. 1B; Supplementary Table S2). Thereafter, H_2_CN_2_ had very little effect on the rate of bud burst. The H_2_CN_2_-treated buds did, however, show an interesting developmental trend; a moderate but progressive increase in the BB_50_ from January (mid-summer) through to August (end winter), from BB_50_ ~30 d to 70 d, before an abrupt decline ~20 d in September, which was shortly before the time of natural bud burst in the field (Fig. 1B). The trend was observed in both the 2015 and 2016 seasons (Fig. 1B; Supplementary Table S2).

**Fig. 2.**
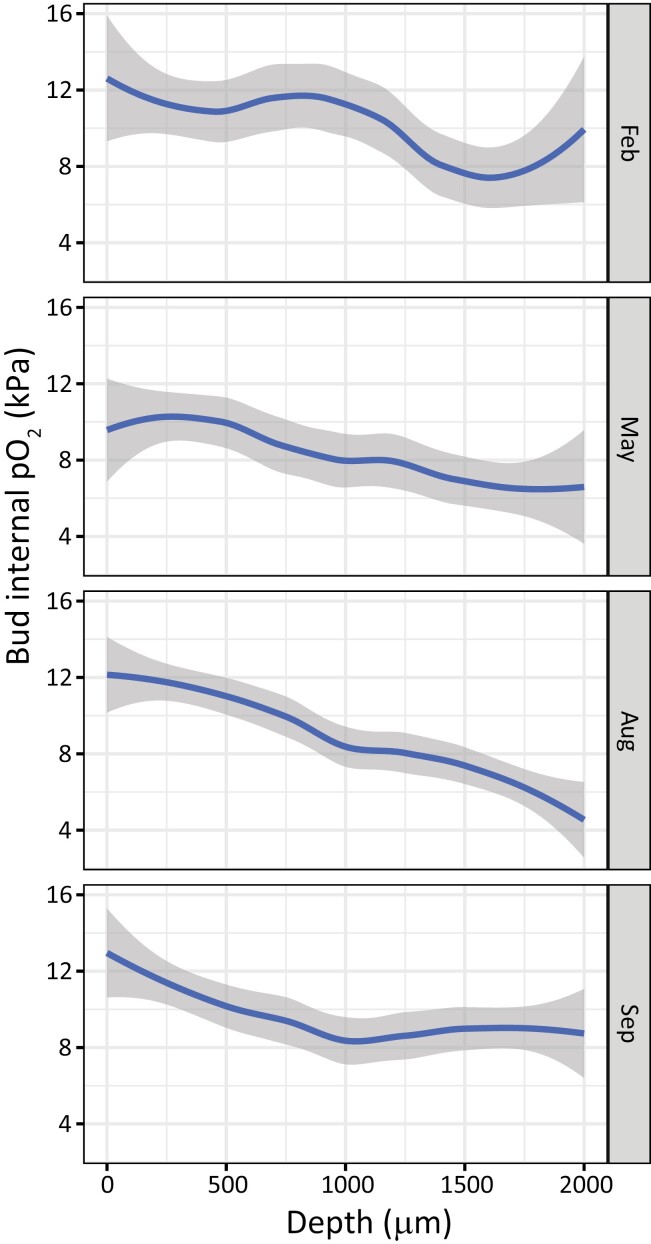
Tissue oxygen partial pressure (pO_2_) profiles in grapevine buds sampled from summer to spring in the Margaret River region of Western Australia in 2015 and 2016. Buds were collected over two consecutive seasons (2015 and 2016), *n*=6 individual buds, with the exception of February (2016 only, *n*=3). Sample dates (Feb, May, Aug, and Sep) are shown in Fig. 1A. Data show the pO2 from immediately within the outer scales (0 μm depth) to the region of the meristematic core of the primary bud (2000 μm). Atmospheric oxygen is ~21 kPa pO_2_. The plot represents a regression curve with 95% confidence intervals.

Matching bud material was used to determine hydration levels, respiration rates, and internal tissue oxygen status. The hydration levels of buds sampled in late January (mid-summer) was ~55 gH_2_O 100 g^–1^ FW (Fig. 1C; Supplementary Table S2). Hydration levels initially declined quite rapidly until April, then more gradually towards a point of inflection at 40 gH_2_O 100 g^–1^ FW by July, and increased thereafter. The O_2_ consumption and CO_2_ production rates remained high until the end of February, before declining rapidly and in parallel by ~2- to 3-fold by April. The gas exchange rates then declined more gradually to reach a minimum during May/June, and increased thereafter (Fig. 1D; Supplementary Table S2). We expected that changes in respiration and hydration may also influence the pO_2_ within buds. Figure 2 shows a moderate but consistent decline in the pO_2_ in the meristematic region of the bud (~1500–2000 μm) from February through to August, before returning to a more normoxic state by September.

Taken together, the data show a remarkable seasonal dynamic of dormancy, with a pronounced but transient peak during late summer/early autumn. This behaviour was not reflected by changes in respiration or hydration levels of the buds. Hydration levels began to decline before changes in dormancy and respiration. The decline in respiration also appeared to precede the decline of dormancy in autumn; however, the rate at which dormancy declined was more rapid. The respiration rate then began to increase in early winter, prior to the increase in hydration levels. Natural bud burst occurs in early to mid-spring, and there was an abrupt increase in hydration at this point. Internal tissue oxygen status was less informative, although there appeared to be a major transition in August, with oxygen levels at the core of the bud becoming more hypoxic, at the time when respiratory oxygen consumption reached a peak prior to natural bud burst in September (Fig. 2).

### Relationship of dormancy and physiology to climate indices

Site weather data were obtained for the 2 years of the study (Supplementary Fig. S3; Supplementary Table S2). The maximum daylength was ~14 h at the summer solstice, which had declined by ~1 h by the earliest sample point 4 weeks later in late January. The minimum daylength was ~10 h at the winter solstice. Temperature maxima/minima ranged from ~28/12 °C in January to ~16/5 °C in July. Rainfall data were also collected, showing a temperate pattern of rain falling predominantly during the winter months (Supplementary Table S2). While the study vineyard site was not irrigated, there was 78 mm and 170 mm of rain in the January to March period of 2015 and 2016, indicating adequate water availability during the drier summer months.

The accumulated chilling in the vineyard was calculated according to three contemporary models (Supplementary Fig. S3). Chilling began to accumulate from April onwards (~100 d post-solstice), at a near linear rate towards and beyond the time of natural bud burst in the field in September (~270 d post-solstice). The chill summation was similar for both Utah models (>1000 units) and ~260 units according to the base 7.2 °C calculation (Supplementary Fig. S3C).

### Differential transcriptome analysis

**Fig. 3.**
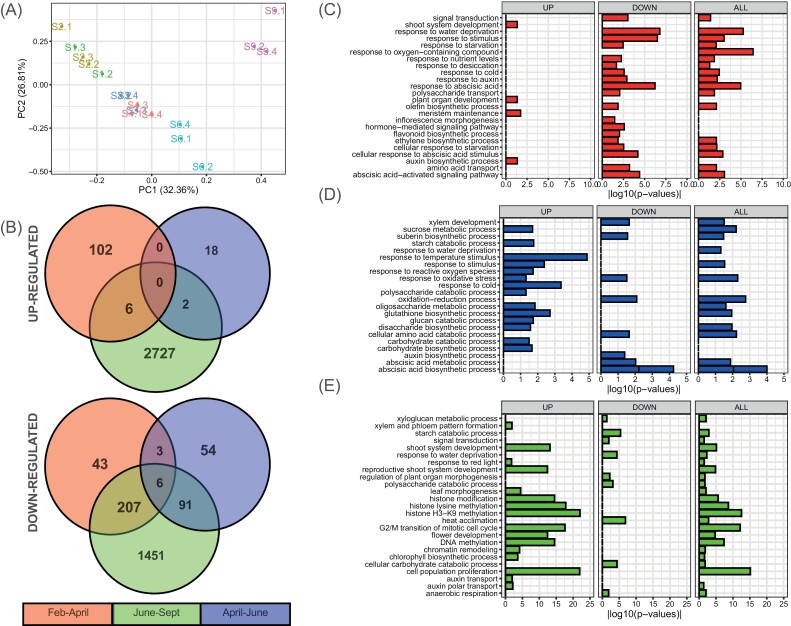
PCA plot of normalized count data, and Venn diagrams and functional enrichment analysis of differentially expressed genes (DEGs) from grapevine buds collected at different stages of dormancy transition February to April, April to June, and June to September. (A) PCA of normalized read counts (S1, January; S2, February; S3, March; S4, April; S6, June; S9, September). (B) Venn diagrams indicating the number of significant (FDR ≤0.01, log_2_FC |1|) DEGs across three comparisons and the overlap between each set of genes separated into up- and down-regulated genes. Horizontal bar plots of selected functional categories significantly enriched (*P*≤0.05 using the hypergeometric test) in the developmental comparisons February–April (C), April–June (D), and June to September (E). Selection was based on relevance to the literature amongst the most significant; complete GO enrichment data are presented in Supplementary Table S4. Three different functional enrichment analyses were performed for each comparison, where ‘ALL’ (up- plus down-regulated genes), ‘UP’ (up-regulated genes), and ‘DOWN’ (down-regulated genes) refer to the list of genes assessed.

RNA extracted from buds collected at six time points in 2015 were sequenced. After pre-processing, normalized log_2_CPM data were used to generate a principal component analysis (PCA) plot, showing that replicates of each time point largely clustered together, and that the September (S9) samples formed the most distinct cluster (Fig. 3A). The first component (32.36%) seemed to be largely affected by developmental age, while the second component (26.81%) appeared to be linked to metabolic activity and degree of hydration (Figs 1, 3A). A similar discrimination along developmental trends was reported previously (Diaz-Riquelme *et al.*, 2012). Differential expression analysis showed very few DEGs between January–February and February–March time points, while PCA showed that February and March clustered together (Fig. 3A). Considering this, and guided by the dormancy, respiration, hydration, and chilling data (Fig. 1; Supplementary Fig. S3), we chose to refine the comparisons to three: (i) February to April (~40–95 d post-solstice), representing a rapid decline from the dormancy peak and a parallel decline in respiration and hydration; (ii) April to June (~95–175 d post-solstice), representing the period prior to winter, accompanied by the minimum hydration and respiration rates, and the onset of chilling accumulation; and (iii) June to September (~175–240 d post-solstice), representing the period during winter, accompanied by the resumption of hydration and respiration and the majority of chilling accumulation. Following differential expression analysis, annotations were assigned to the core set of DEGs against the *V. vinifera* 12X v.2.1 annotation file, and functional enrichment was performed based on GO analysis.

A total of 4490 DEGs were identified across the three stages of dormancy transition (Supplementary Table S3), which represents ~10% of the predicted genes in the *V. vinifera* genome (Canaguier *et al.*, 2017). A Venn diagram of DEGs revealed that the June–September transition was the most discriminating, as over half of all DEGs (2727) were uniquely up-regulated and one-third (1451) uniquely down-regulated during this transition (Fig. 3B). Few genes were consistently up-regulated between successive transitions; no genes were consistently up-regulated throughout the experiment and only six genes were consistently down-regulated (Fig. 3B). These data suggest stage-specific gene expression profiles during the subsequent developmental transitions. Functional enrichment analysis showed that genes involved in several biological processes, including hormone signalling, cell proliferation, morphological development, and epigenetic regulation, were highly regulated during the time course (Fig. 3C–E). Figure 3C–E shows a selection of GO dats for biological processes; comprehensive GO enrichment data are presented in Supplementary Table S4.

### The transition from summer to autumn

The February to April transition represented a 4-fold decline in BB_50_ and parallel declines in hydration and cellular respiration (Fig. 1B–D). The GO enrichment, together with the DEG data revealed a pronounced decline in the expression of response to stimuli functions, including abiotic, hormone, and nutrient stimuli (Fig. 3C; Supplementary Tables S3, S4). Expression profiles and unique identifiers of all genes subsequently described are shown in Supplementary Table S5.

Down-regulated abiotic response genes included four homologues of *GALACTINOL SYNTHASE* as well as a *RAFFINOSE SYNTHASE* and *STACHYOSE SYNTHASE*, which collectively encode functions to synthesize raffinose family oligosaccharides (RFOs; Supplementary Table S5). *VACUOLAR INVERTASE 2* (*VI2*), involved in sucrose catabolism, and a sugar transporter *SWEET17* (previously known as *NODULIN MtN3*) were down-regulated, as were an *AMMONIUM TRANSPORTER 2* and an *AMINO ACID PERMEASE*, indicative of a transcriptional down-regulation of nutrient transport functions from February to April. Genes involved in ABA biosynthesis and signalling were down-regulated: two *9-CIS-EPOXYCAROTENOID DIOXYGENASES*, and *MOTHER OF FT AND TFL1* (*MFT*), together with four *LATE EMBRYOGENESIS ABUNDANT* and two homologues of *SENESCENCE-ASSOCIATED GENE 101*. Genes encoding functions that generate or process reactive oxygen species (ROS) were also down-regulated during this transition

Against this, a small number of functions assigned to phyllome development were significantly enriched in the GO data, and up-regulated during this transition (Supplementary Table S4). For example, a *DORNROSCHEN-like* (*DRN-LIKE*/*ESR2*) ethylene response factor (ERF) transcription factor, two homologues of *ASYMMETRIC LEAVES 1*, and two gibberellic acid (GA)-responsive genes were up-regulated (Supplementary Table S5). Interestingly *DRN-LIKE* is the most homologous grapevine gene to the poplar *EARLY BUD-BREAK 1* (*EBB1*), which is a positive regulator of bud burst (Yordanov *et al.*, 2014; Busov *et al.*, 2016). Taken together, these data indicate that the February condition, where the time to BB_50_ was maximal, was highly regulated, with relatively high expression of genes functioning in responses to abiotic stimuli, ABA, and ROS. The trend to a decline in ABA synthesis and responses may, however indicate that ABA levels remained high and were in the process of down-regulation.

### The transition from autumn to winter

By comparison with the previous transition, April to June represented quite modest declines in the BB_50_, hydration, and the rate of respiration (Fig. 1). The number of genes differentially regulated was also the least of each of the transitions, and the majority were down-regulated (Fig. 3B; Supplementary Table S3). Nevertheless, GO analysis revealed a prominent enrichment of carbohydrate metabolism, namely sucrose metabolism, starch catabolism, polysaccharide catabolism, oligosaccharide metabolism, glucan catabolism, disaccharide biosynthesis, and carbohydrate catabolism and biosynthesis. None of these GO terms was enriched in the other two transitions (Fig. 3), suggesting a specific regulation of carbohydrate metabolism during the autumn to winter transition. GO analysis also revealed that responses to temperature, cold, and oxidative stress/ROS were more enriched in June, relative to April (Fig. 3D; Supplementary Table S4).

Transcripts coding for the ABA synthetic enzymes *NCED6* and *ABA DEFICIENT 2* (*ABA2*) were down-regulated, as were genes coding for the synthesis of RFOs, which were down-regulated in the previous transition (Supplementary Table S3, S5). A *PEROXIDASE 1* and *GLUTATHIONE PEROXIDASE 8* were representative of declines in ROS processing functions, although a *ROXY1* thioredoxin superfamily protein was up-regulated. *γ-GLUTAMYLCYSTEINE SYNTHETASE* (*ECS1*), which codes for the first committed step of glutathione synthesis, was also up-regulated. Up-regulation of temperature-regulated genes was represented by two homologues of the *COR27* cold-regulated gene. Together, these findings are consistent with acclimation to abiotic stress and desiccation, and a more metabolically quiescent state than the preceding phase.

### The transition from winter to spring

The final developmental comparison of June to September was accompanied by a relatively modest decline in the BB_50_; however, hydration, the rate of respiration, and tissue oxygen status increased markedly, as did photoperiod and the cumulative exposure to chilling (Figs 1, 2; Supplementary Fig. S2). By this stage, buds had not burst naturally in the field, although bud burst was imminent. The number of uniquely up- or down-regulated genes was the greatest of any transition (Fig. 3B). The GO analysis showed a strong enrichment of functions assigned to DNA replication and epigenetic modification in this transition, such as histone modification, histone lysine methylation, histone H3-K9 methylation, G_2_/M transition of mitotic cells, DNA methylation, and chromatin remodelling (Fig. 3E; Supplementary Table S4). In addition, GO analysis revealed a significant enrichment of transcripts involved in shoot and vascular system development. Numerous functions were unique or regulated in an opposite direction to changes of previous transitions.

Four homologues of *CHROMOMETHYLASE 2* and *3*, a *METHYLTRANSFERASE 1* (*MET1*), and *DECREASE DNA METHYLATION 1* (*DDM1*) were up-regulated in September (Supplementary Table S5). In addition, *ARABIDOPSIS TRITHORAX-RELATED PROTEIN* (*ATXR*) *5* and *ATXR6*, two genes encoding a nucleolar histone methyltransferase-related protein, and a histone deacetylase were up-regulated, as were numerous genes coding for histone subunits. *VERNALIZATION 1* (*VRN1*), *VERNALIZATION 3-LIKE* (*VEL1*), and other related epigenetic factors are also outlined below, while cell cycle genes are outlined in a later section.

ABA- and stress-responsive genes were widely down-regulated. These included homologues of *NCED4*, *LEA4-5*, dehydrin *XERO1*, three cold-regulated *COR27*, and four PP2C genes, namely *ABA INSENSITIVE 1* (*ABI1*), *ABA-HYPERSENSITIVE GERMINATION 3* (*AHG3*). and two *HIGHLY ABA-INDUCED* genes (*HAI2* and *HAI3*; Supplementary Table S5). Two of the *COR27* genes were previously up-regulated from April to June. Homologues of SNF1-related protein kinase genes were down-regulated (*SNRK2.6*, *SNRK3.14*, *SNRK3.8*, *AKINBETA 1*, and *KING1*), as were genes coding for dormancy-associated proteins DELAY OF GERMINATION 1 (DOG1) and DORMANCY ASSOCIATED PROTEIN 1 (DRM1). Against this, genes encoding auxin transport and signalling functions were predominantly up-regulated. These included two homologues encoding the auxin efflux carrier *PIN-FORMED 1* (*PIN1*), plus *PIN2*, *PIN5*, and *PIN6*, together with the auxin receptor *TRANSPORT INHIBITOR RESPONSE 1* (*TIR1*) and a number of auxin-responsive factor proteins. Cytokinin signalling genes were also up-regulated, including a number of homologues of *ARABIDOPSIS RESPONSE REGULATOR* family genes. Meanwhile GA- and ethylene-related functions were differentially regulated. GA biosynthetic genes were up-regulated, as was a homologue of the DELLA-degrading F-box protein SLEEPY2, while a GA receptor (*GID1B*) and two genes of GASA domain proteins were down-regulated. Similarly, the ethylene biosynthetic gene *ACC OXIDASE 1* was up-regulated while *ETR1* and *EIN3* were down-regulated. Together these indicate a finely controlled transition of hormone synthesis and processing during bud development between June and September, with a considerable decline in the influence of ABA against an increase in that of auxin- and cytokinin-dependent functions.

Together with the down-regulation of cold-regulated *COR27*, the up-regulation of *VRN1*, *VEL1*, and *DROUGHT SENSITIVE 1* and changes in metabolic functions were consistent with acclimation following stress (Supplementary Table S5). A number of *AMYLASE* genes were down-regulated, while three homologues of *PHOSPHOENOLPYRUVATE CARBOXYKINASE 1*, three *TREHALOSE-6-PHOSPHATE SYNTHASE* (*TPS*) genes, *TREHALOSE-6-PHOSPHATE PHOSPHATASE*, three *SUCROSE SYNTHASE* (*SUSY*) genes, and *SEED IMBIBITION 1* and *2*, which encode raffinose synthases were up-regulated. Important genes involved in facilitating transmembrane sugar and nitrogen transport were up-regulated. These included *CELL WALL INVERTASE 1, SWEET 17*, and *ERD SIX-LIKE 1*, a homologue of a stress-inducible monosaccharide transporter. Two homologues of *AMINO ACID PERMEASE 2* and a nitrate transporter *NRT1:2* were up-regulated. These reflect a transcriptional up-regulation of gluconeogenesis, sucrose, trehalose, and raffinose metabolism and transport functions during this transition.

Cell wall, pectin, and cellulose metabolism was widely regulated during this transition, through up-regulation of a number of genes encoding cellulose and glucan synthases, pectin methylesterase, and invertase/pectin methylesterase inhibitors (Supplementary Table S5).

Genes encoding redox-related functions were largely up-regulated, including homologues of *RBOHD*, *ROXY1*, and *ROXY2* (Supplementary Table S5). Ascorbate synthesis was positively regulated through *l**-GALACTONO-1,4-LACTONE DEHYDROGENASE* (*GLDH*), as was cysteine through *O-ACETYLSERINE (THIOL) LYASE* (*OAS-TL*), while glutathione synthesis was mildly down-regulated (*ECS1*). Response to hypoxia was also evident; homologues of 10/49 conserved hypoxia response genes were regulated only at this final transition (Mustroph *et al.*, 2009; Supplementary Table S5). These included *ACC OXIDASE 1*, *LOB DOMAIN-CONTAINING PROTEIN 41* (*LBD41*), *SUSY4*, *PLANT CYSTEINE OXIDASE 1* (*PCO1*), and a homologue of *RBOHD* (mentioned above), which were strongly up-regulated, consistent with the function in hypoxic response. Also up-regulated were *RELATED TO AP 2.3* (*RAP2.3*) and *LITTLE ZIPPER 2* (*ZPR2*), which have demonstrated functions in hypoxia (Weits *et al.*, 2019).

Numerous genes encoding cell identity, meristem, and flowering functions were up-regulated in addition to those mentioned above (*VRN1*, *VEL3*, *ZPR2*, and *LBD41*; Supplementary Table S5). These include multiple homologues of *WUSCHEL-RELATED HOMEOBOX* proteins (*WOX1*, *3*, *4*, and *9*), *CLAVATA 1* (*CLV1*), *CLAVATA3/ESR-RELATED 44* (*CLE44*), and *SHOOT MERISTEMLESS* (*STM*).

The broad functions represented in the transcriptional changes from June to September are consistent with a post-acclimation reorganization of nuclear, metabolic, and cellular structure and function. The prominence of redox- and hypoxia-responsive genes may reflect a relationship to chromatin regulation and differentiation.

### Comparison of dormancy and bud burst data

In earlier studies we have identified a number of developmentally and light-regulated transcripts during the early transition towards bud burst (Meitha *et al.*, 2018; Signorelli *et al.*, 2018). A comparison of those DEG datasets with the present set revealed 80 genes that were differentially regulated in all studies (Supplementary Table S5). Of these, only two were not differentially regulated in the transition from winter to spring here. There was considerable agreement between those genes regulated between winter and spring and those light regulated in the first 144 h of bud burst. This included the down-regulation of *DRM1* and a SNF1-related protein kinase gene *KING1*, and up-regulation of *CRYPTOCHROME 3* (*CRY3*), glutamyl-tRNA reductase *HEMA1*, *GENOMES UNCOUPLED 4* (*GUN4*), and *PROTOCHLOROPHYLLIDE OXIDOREDUCTASE C* (*PORC*). By corollary, those DEGs that were unique to the data reported here provide insight into the molecular regulation of the onset and maintenance of bud dormancy.

### *Cis*-regulatory element enrichment identifies common and unique stress and developmental gene regulation

**Fig. 4.**
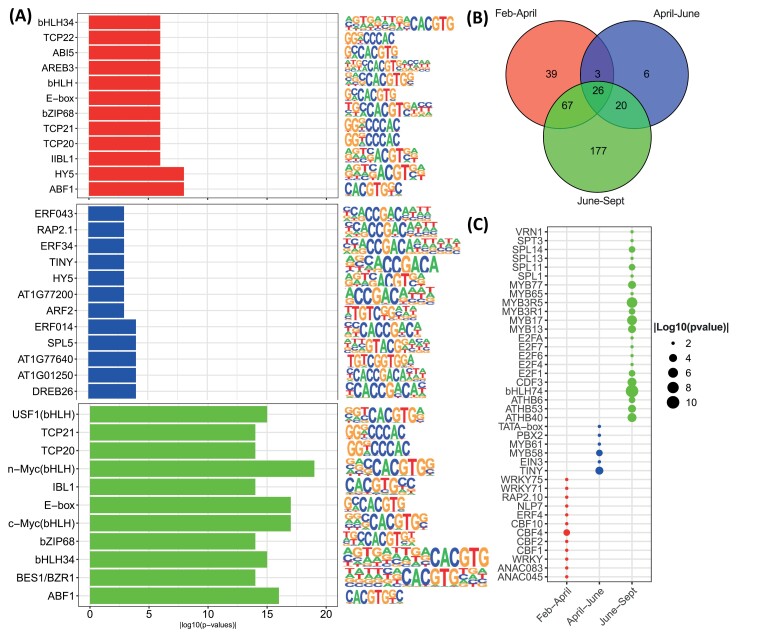
Enrichment analysis of known *cis*-regulatory binding motifs identified in promoter region DEGs (FDR ≤0.01, log_2_FC |1|) at different stages of bud dormancy transition for the comparisons, February to April, April to June, and June to September. (A) Bar plots showing the 10 most significantly enriched motifs (lowest *P*-value) for the comparisons, February–April (red), April–June (blue), and June–September (green). (B) Venn diagram discriminating the common and unique motifs in the three comparisons. (C) A scatter plot of selected unique motifs found in each comparison.

To gain further insight into the prevailing transcriptional control during dormancy transitions, we carried out enrichment analysis for known and *de novo cis*-regulatory motifs in the 1.2 kb upstream region of the DEGs (Fig. 4; Supplementary Table S6). A remarkably large proportion of the total number of enriched motifs were common to all transitions (Fig. 4B). Figure 4A shows the most significant (lowest *P*-value) motifs identified, and Fig. 4C shows a selection of the unique motifs identified at each transition. The TEOSINTE BRANCHED1, CYCLOIDEA, PCF (TCP), and ETHYLENE-RESPONSIVE TRANSCRIPTION FACTOR (ERF) binding sites featured prominently throughout (Fig. 4A). Interestingly four C-REPEAT BINDING FACTOR- (CBF) binding sites were specifically represented in the February to April dataset. Meanwhile the MYB3R and SPL motifs predominated among enriched motifs in the June to September dataset, suggesting a prominent role for the MYB3R and SPL family transcription factors during the final transition preceding bud burst.

### Core cell cycle genes, water relations, and diverse roles of ERF family genes during dormancy transitions

**Fig. 5.**
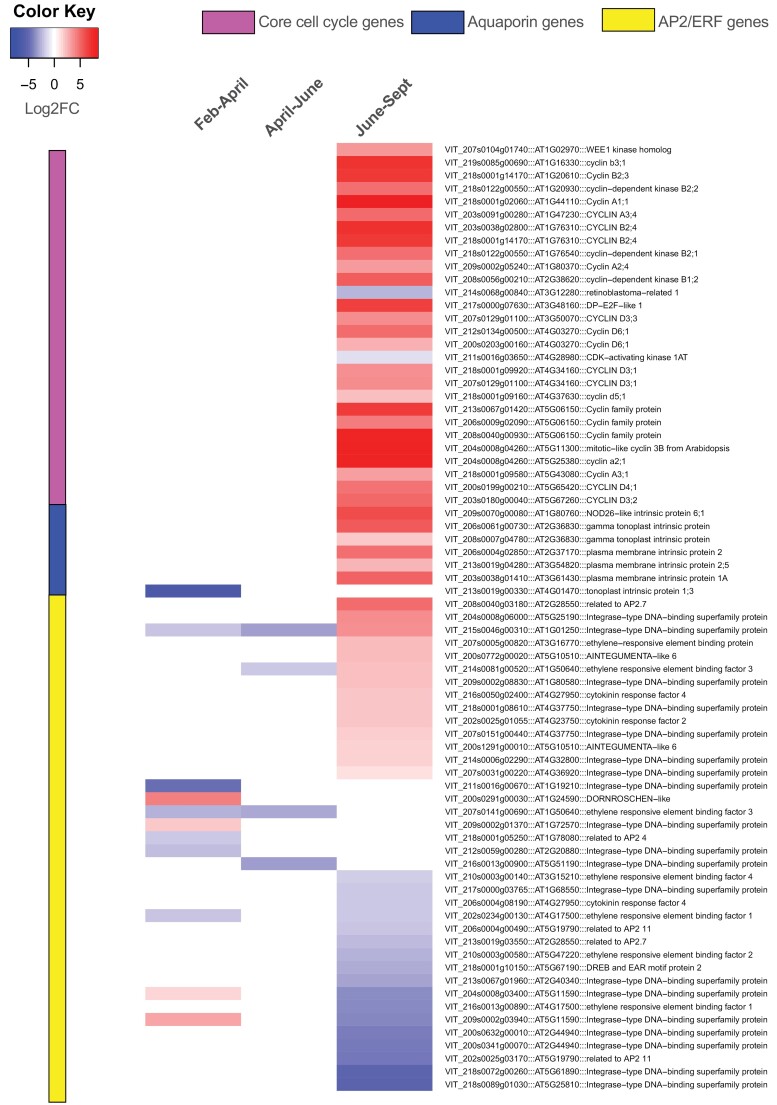
Differential expression of putative cell cycle, aquaporin (water relations), and APETALA2/ethylene responsive factor (AP2/ERF) genes among the DEGs (FDR ≤0.01, log_2_FC |1|) during different stages of bud dormancy transition February to April, April to June, and June to September. Homologues of core cell cycle genes of Arabidopsis (Vandepoele *et al.*, 2002) are shown in the upper panel of the heatmap (adjacent to the purple column). Homologues of the AP2/EREBP family of plant transcription factors of Arabidopsis (Riechmann and Meyerowitz, 1998) are shown in the blue panel of the heatmap. Homologues of aquaporin genes of Arabidopsis (Ward, 2001) are shown in the lower three panels of the heatmap (orange–yellow).

Our earlier studies have indicated that oxygen and ethylene signalling (Meitha *et al.*, 2015, 2018) and cell–cell transport (Signorelli *et al.*, 2020) may have key regulatory roles in bud development. In addition, we have reviewed the role of cell cycle regulation as a master or slave of dormancy transitions (Velappan *et al.*, 2017). We thus sought to evaluate the transcriptional data here for evidence in support of these functions. Previous studies have identified a set of 61 core cell cycle genes in Arabidopsis (Vandepoele *et al.*, 2002), 138 members of the APETALA2/ethylene-responsive element-binding protein (AP2/EREBP) family of plant transcription factors (Riechmann and Meyerowitz, 1998), and 33 aquaporin genes (Ward, 2001) in Arabidopsis. We used the *Vitis* 12X v.2.1 annotation file to identify a set of *Vitis* homologues of these genes within our set of DEGs (Supplementary Table S7). We identified 28 cell cycle- and seven aquaporin-related genes (purple and blue panel) which were all up-regulated during the final transition preceding bud burst (June to September; Fig. 5; Supplementary Table S7). The dehydration-responsive element-binding protein (DREB) subfamily of the AP2/EREBP family were largely down-regulated from June to September (Fig. 5). As mentioned above, the grapevine homologue of the poplar *EBB1* (*DRN-LIKE*) was strongly up-regulated in the February to April transition and unchanged thereafter, which is not consistent with the proposed EBB1 function in other species (Yordanov *et al.*, 2014; Busov *et al.*, 2016). Other AP2/EREBP family genes had no definitive expression pattern, which suggests functional diversity amongst this family of transcription factors.

## Discussion

The depth and seasonal dynamics of dormancy (BB_50_) reported here for cv. Merlot (33°47ʹS) correlated very well with those reported for cv. Merlot (44°50ʹN) and cv. Carignan (43°36ʹN) (Pouget, 1963; Nigond, 1967), and reasonably well with cv. Thompson Seedless (33°34ʹS) (Rubio *et al.*, 2016, 2019; Parada *et al.*, 2016), cv. Chardonnay, and cv. Cabernet Sauvignon (46°17ʹN) (Camargo Alvarez *et al.*, 2018), although the magnitude differed. Trends in respiration data also correlated well with cv. Merlot (44°50ʹN) (Pouget, 1963) and cv. Thompson Seedless (33°34ʹS) (Parada *et al.*, 2016). However, while Parada *et al.* (2016) reported an inverse relationship between respiration and BB_50_, our data show that respiration was not a good predictor of the potential BB_50_, neither was water content (Fig. 1), in particular during summer and autumn. Collectively, the data presented herein and by Pouget (1963) and Nigond (1967) reveal a disconnect between growth quiescence (syn. dormancy) and metabolic quiescence during the early stages of dormancy in summer and autumn. According to accepted horticultural definitions (Lang *et al.*, 1987), this period represents the onset of endodormancy. However, according to more binary definitions, as related to the capacity to resume growth, the physiology of buds in late summer is quite unexplained. In contrast, the growth physiology of buds collected from late autumn onwards, following the commencement of chilling, are congruent with the contemporary literature on grapevine (Rubio *et al.*, 2016; Zheng *et al.*, 2018*a*). The question of why this pronounced disconnect during late summer was not observed by other studies cited earlier may reflect genotypic differences which remain to be explored. Here we focus the discussion on the novel insights made possible by the transcriptome data that support hypothesis development. These include the role of photoperiod and ABA during the early stages of dormancy, and roles of light, oxygen, and hypoxia in directing chromatin regulation that governs late development during winter and spring.

### ABA-dependent processes govern early seasonal changes in transcription

As described above, ABA plays important roles in the acquisition of dormancy, as well as cold and desiccation tolerance. In poplar, the function of ABA in the onset of dormancy is downstream of photoperiod responses (Tylewicz *et al.*, 2018). In our data, the transition from summer to autumn accompanied a strong down-regulation of genes coding for ABA biosynthesis and responses, notably the synthesis of RFOs (Fig. 3C; Supplementary Table S5). Additional ABA and RFO synthetic genes were down-regulated at the subsequent stage from autumn to winter, while ABA signalling genes were down-regulated in the final transition to spring (Fig. 3D–E; Supplementary Table S5). In addition, the motif enrichment analysis indicated that ABA may mediate photoperiod responses in the onset of dormancy in grapevine (Fig. 4).

ABA positively regulates the synthesis of RFOs in a number of species, which in turn play important roles in development, energy homeostasis, desiccation tolerance, and response to oxidative stress (Nishizawa *et al.*, 2008; Nishizawa-Yokoi *et al.*, 2008;Urano *et al.*, 2009; Sengupta *et al.*, 2015). In glasshouse-grown vines, *in situ* buds of daylength-sensitive *V. riparia* accumulated ABA content up to 21 d after exposure to short days, followed by a mild decline, while raffinose and trehalose metabolite levels did not accumulate until 28–42 d, when the buds were deemed to be dormant (Fennell *et al.*, 2015). Four homologues of *GALACTINOL SYNTHASE* were up-regulated at the dormant stage (28–42 d) in the short day-grown *V. riparia* compared with those grown in long days (Supplementary Table S5). The *V. riparia* data appear to be consistent with those seen in apple and other temperate/boreal species, where homologues of *GALACTINOL SYNTHASE* showed strong circannual rhythms, that were paralleled by the concentration of galactinol and raffinose (Cox and Stushnoff, 2001; Derory *et al.*, 2006; Pagter *et al.*, 2008; Ibánez *et al.*, 2013; Falavigna *et al.*, 2018). Wang *et al.* (2020) reported an increase in concentration of galactinol and stachyose in the explants following application of exogenous ABA. In the absence of exogenous ABA, however, there was little temporal change in sugar and oligosaccharide content (Wang *et al.*, 2020).

As described above, the induction of ABA catabolism by the overexpression of *VvA8H-CYP707A4* accelerated bud burst, whether forced in autumn, winter, or spring (Zheng *et al.*, 2018*a*). While homologues of *A8H-CYP707A* were not differentially regulated, our data were consistent with a decline in ABA signalling responses in the transition to spring, including the down-regulation of *XERO1*, *DOG1*, *HAI2*, *HAI3*, *DRM1*, and genes coding for SNF1-related protein kinases (Supplementary Table S5). These data suggest that ABA and RFO levels were already high in the buds of our earliest time point and played an important role in the regulation of respiration, desiccation, acclimation to stress, and physiological quiescence during the transition to autumn.

In addition, we identified specific enrichment of CBF-binding sites in the 5ʹ-untranslated region of DEGs of the February to April transition (Fig. 4C; Supplementary Tables S5, S6). CBFs are widely involved in response to abiotic stress, notably freezing tolerance and cold acclimation; however, functions or regulation of different members appear to have diverged (Novillo *et al.*, 2007). Three CBFs of grapevine were found to possess 20–29 additional amino acids and three serine stretches that were not characteristic of CBFs of other species (Xiao *et al.*, 2006), and were also absent from a fourth grapevine CBF (CBF4) (Xiao *et al.*, 2008). Each *Vitis* CBF had somewhat distinct expression patterns, responses to abiotic cues including ABA, and ability to trans-activate C-repeat elements. The relationship between the CBF enrichment and the dormancy dynamics here deserves further attention. For example, expression of *CBF1–CBF3* in Arabidopsis is influenced by photoperiod in warmer temperatures, such that phytochrome B signalling represses CBF expression (Lee and Thomashow, 2012). Furthermore, ELONGATED HYPOCOTYL 5 (HY5) directly promotes the *ABI5* expression in seedlings (Chen *et al.*, 2008). HY5, together with ABI5 and PHYTOCHROME INTERACTING FACTOR (PIF) motifs, was enriched in our motif analysis of the February to April transition (Supplementary Table S6). Taken together, this warrants further investigation of the influence of photoperiod on gene expression, ABA function, and dormancy in cultivated grapevine.

### Light and temperature responses implicate a role for blue light signalling in the resumption of growth

In our data, regulation of *CRY3*, *HEMA1*, *GUN4*, *PORC*, and the *COR27* homologues was among the most upstream transcriptional response to light and temperature (Supplementary Table S5). *CRY3* encodes a cryptochrome receptor for blue and UVA light. Together with other photoreceptors, it positively regulates photomorphogenesis via the HY5 transcription factor (Gangappa and Botto, 2016). HY5 directly regulates other light signalling genes, including those coding for the synthesis of chlorophyll, as seen prominently here: *HEMA1*, *GUN4*, *PORC*, and a number of light-harvesting complex genes. *HY5*, together with *CRY3*, *HEMA1*, *GUN4*, and *PORC*, was light regulated during bud burst (Meitha *et al.*, 2018; Signorelli *et al.*, 2018), but was not observed in the present data. In addition, we observed down-regulation of *DRM1* and *KING1* in the transition to spring here and in light-grown buds during bud burst (Meitha *et al.*, 2018; Signorelli *et al.*, 2018). Both *DRM1* and *KING1* genes in Arabidopsis are repressed by blue light (Jiao *et al.*, 2003; Kleine *et al.*, 2007), and blue light and cytokinins function together in photomorphogenic processes during bud outgrowth (Roman *et al.*, 2016; Signorelli *et al.*, 2018).

The *COR27* and *COR28* homologues are key integrators of light and temperature cues in Arabidopsis (Fowler and Thomashow, 2002). The Arabidopsis COR27 and COR28 proteins are stabilized by blue light and directly interact with HY5 in transcriptional regulation of photomorphogenesis (Li *et al.*, 2020). These proteins function downstream of CIRCADIAN CLOCK ASSOCIATED 1 (CCA1) and CONSTITUTIVE PHOTOMORPHOGENIC 1 (COP1) but upstream of floral regulators, as well as playing a role in the development of freezing tolerance (Li *et al.*, 2016, 2020). The prominent regulation of three *COR27* homologues from autumn through to spring is thus consistent with a regulatory function of blue light signalling via cryptochromes.

### Redox and hypoxia signalling may play important roles in structural remodelling, DNA synthesis, and DNA repair during bud development

There was prominent regulation of genes coding for redox homeostasis and carbon metabolism at each transition (Fig. 3C–E; Supplementary Table S5). Sugar and nitrogen transporters, and genes coding for gluconeogenesis were prominently down-regulated between summer and autumn, while these functions, together with activities that mobilize sugars from lipids, organic acids, and oligosaccharides, were up-regulated from winter to spring. In the intervening transition from autumn to winter, there was little change, indicating metabolic quiescence. The synthesis of cysteine, glutathione, and ascorbate was strongly regulated in the transition to spring, along with *RBOHD*, *ROXY1*, and *ROXY2*, and other prominent redox processing functions. Moreover, 10 homologues of the 49 Arabidopsis hypoxia response genes (Mustroph *et al.*, 2009) were differentially regulated in the transition from winter to spring, together with *RAP2.3* and *ZPR2* (Weits *et al.*, 2019).

Redox processing plays important metabolic roles, and ROS are intricately involved in cell expansion, cell wall thickening, and the conductivity of plasmodesmata (Gapper and Dolan, 2006; Benitez-Alfonso *et al.*, 2011; Considine and Foyer, 2014). We have previously demonstrated strong temporal and spatial correlation between ROS and lignin abundance during bud burst in grapevine (Meitha *et al.*, 2015), and data here indicate that these processes commence well before the acute transition to bud burst. Developmental hypoxia also plays important roles in meristem functions and metabolic regulation, including during bud burst (Considine *et al.*, 2017; Meitha *et al.*, 2018; Gibbs *et al.*, 2018; Weits *et al.*, 2019, 2020). The pO_2_ data shown here are consistent with developmental control of hypoxia, through metabolic regulation and diffusion constraints via structural changes. It is important to indicate the strong regulation of the nuclear landscape, DNA synthesis, and repair, which was most prominent in the transition towards spring. Redox processing is critical for enabling a competent rhythm during the cell cycle upon imbibition in seeds (de Simone *et al.*, 2017), and regulated ROS synthesis is important for DNA synthesis and repair, and regulation of cell cycle transitions (Tsukagoshi *et al.*, 2010; Velappan *et al.*, 2017).

### Conclusions

The form and phenology of grapevine display considerable plasticity to climate and seasonality. Understanding the relationship between metabolism, cell signalling, and growth physiology is essential to enable changes in practice that accommodate regional climate change and enable increased productivity in marginal, warmer climates. The present data establish a reproducible disconnect between the capacity to grow (syn. dormancy) and metabolic and transcriptional regulation following bud set in summer and autumn.

We observed an extreme resistance of explants collected in late summer to resume growth, which was not consistent with the seasonal dynamics of climate, physiology, or gene expression. Interpretations of the field-state data, however, were consistent with an important regulatory role for ABA in the onset of dormancy and acclimation, and motif analysis of DEGs indicated the potential role of photoperiod in regulating ABA function. Specific gene regulation of light, hypoxia, and redox signalling functions during the transition to spring were accompanied by strong up-regulation of histone and chromatin regulators, together with canonical genes of the cell cycle and meristem identity. Together, this study provides critical insight to the understanding of the regulation of quiescence, and prompts wider investigation of the seasonal and climate dependencies of phenology in grapevine.

## Supplementary data

The following supplementary data are available at *JXB* online.

Fig. S1. Box plots of read counts/effective library sizes before (A) and after (B) normalization and a multidimensional scaling (MDS) plot (C) showing the similarities and dissimilarities between samples.

Fig. S2. Scatterplots of the distribution of means (*x*-axis) and variances (*y*-axis) of each gene showing the dependence between the two (A) before and (B) after Voom is applied to the data.

Fig. S3. Seasonal changes in photoperiod (A), temperature (B), and cumulative chilling (C) in the Margaret River region of Western Australia (2015).

Table S1. RNAseq data statistics describing reads pre- and post-quality control and number of reads that mapped to the genome (effective library size).

Table S2. Bud burst, physiological, and climate data for 2015 and 2016 Merlot buds grown in Margaret River region of Western Australia (33°S latitude).

Table S3. Number of genes differentially expressed (FDR ≤0.01, log_2_FC |1|) in developmental comparisons from grapevine buds sampled from summer to spring in the Margaret River region of Western Australia in 2015.

Table S4. Statistically significant Gene Ontology terms of differentially expressed genes in grapevine buds sampled from summer to spring in the Margaret River region of Western Australia in 2015.

Table S5. A subset of Supplementary Table S3 referring to specific genes outlined in the text.

Table S6. Statistically enriched known *cis*-elements/motifs 1.2 kb upstream of the transcriptional start site (TSS) of differentially expressed genes in grapevine buds sampled from summer to spring in the Margaret River region of Western Australia in 2015.

Table S7. Homologues of cell cycle, aquaporin, and AP2/ERF genes and their expression patterns in the DEG dataset, as used for Fig. 5.

erac001_suppl_supplementary_figures_S1-S3

erac001_suppl_supplementary_table_S1

erac001_suppl_supplementary_table_S2

erac001_suppl_supplementary_table_S3

erac001_suppl_supplementary_table_S4

erac001_suppl_supplementary_table_S5

erac001_suppl_supplementary_table_S6

erac001_suppl_supplementary_table_S7

## Data Availability

Raw RNAseq data files have been submitted to NCBI (BioProject ID PRJNA575976, http://www.ncbi.nlm.nih.gov/bioproject/575796). Physiological data are available from the corresponding author, Michael Considine, upon request.
