# Supplementary material for: The bud dormancy disconnect: latent buds of grapevine are dormant during summer despite a high metabolic rate
Source: J Exp Bot. 2022 Jan 11;73(7):2061–76. doi: 10.1093/jxb/erac001 (PMC8982382; doi:10.1093/jxb/erac001)
Supplement: erac001_suppl_supplementary_figures_S1-S3 [file erac001_suppl_supplementary_figures_s1-s3.pdf]

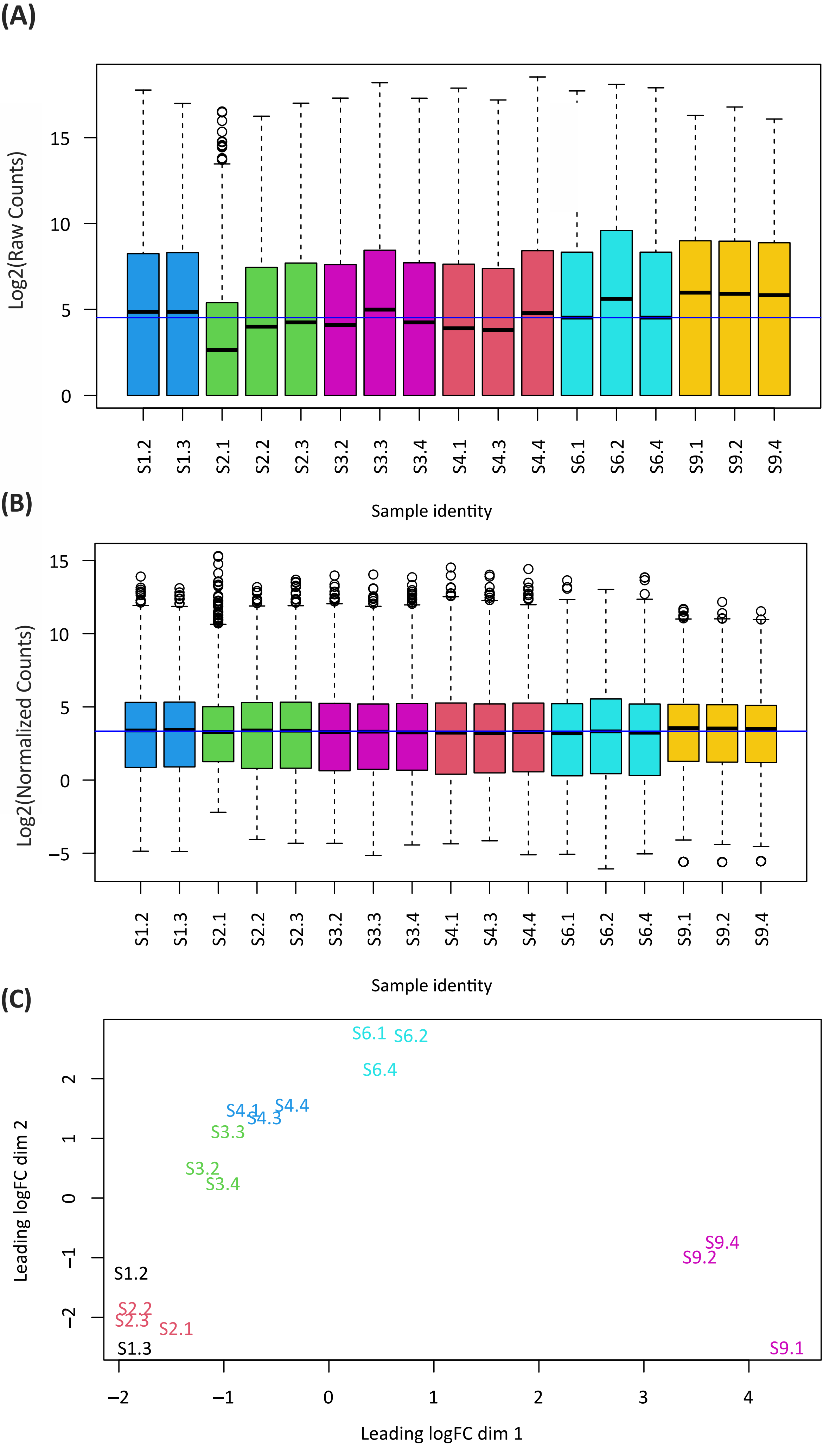

Supplemental Figure S1. Box plots of read counts/effective library sizes before (A) and after (B) normalization and a multi-dimensional scaling (MDS) plot (C) showing the similarities and dissimilarities between samples

(A)

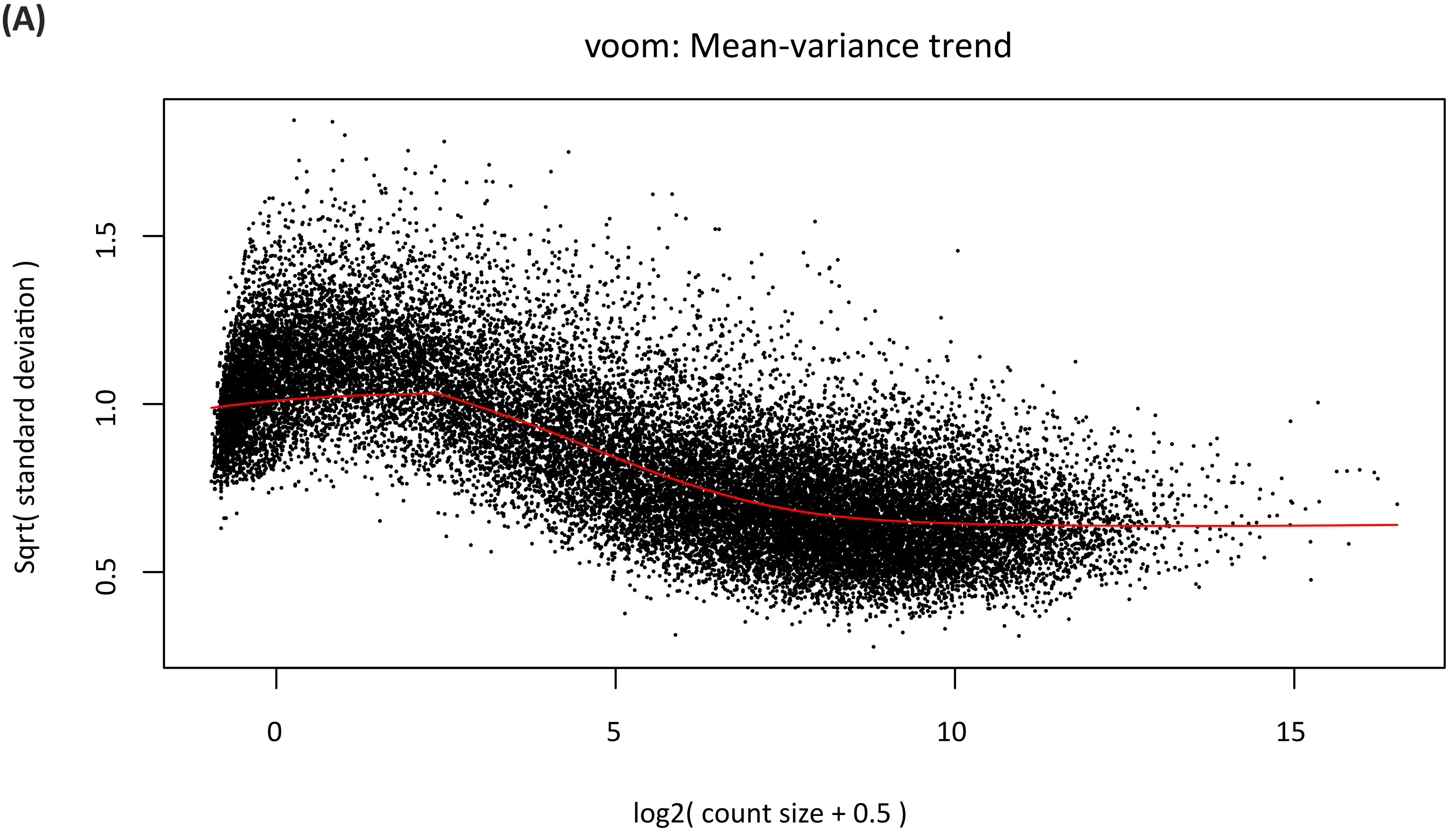

(B)

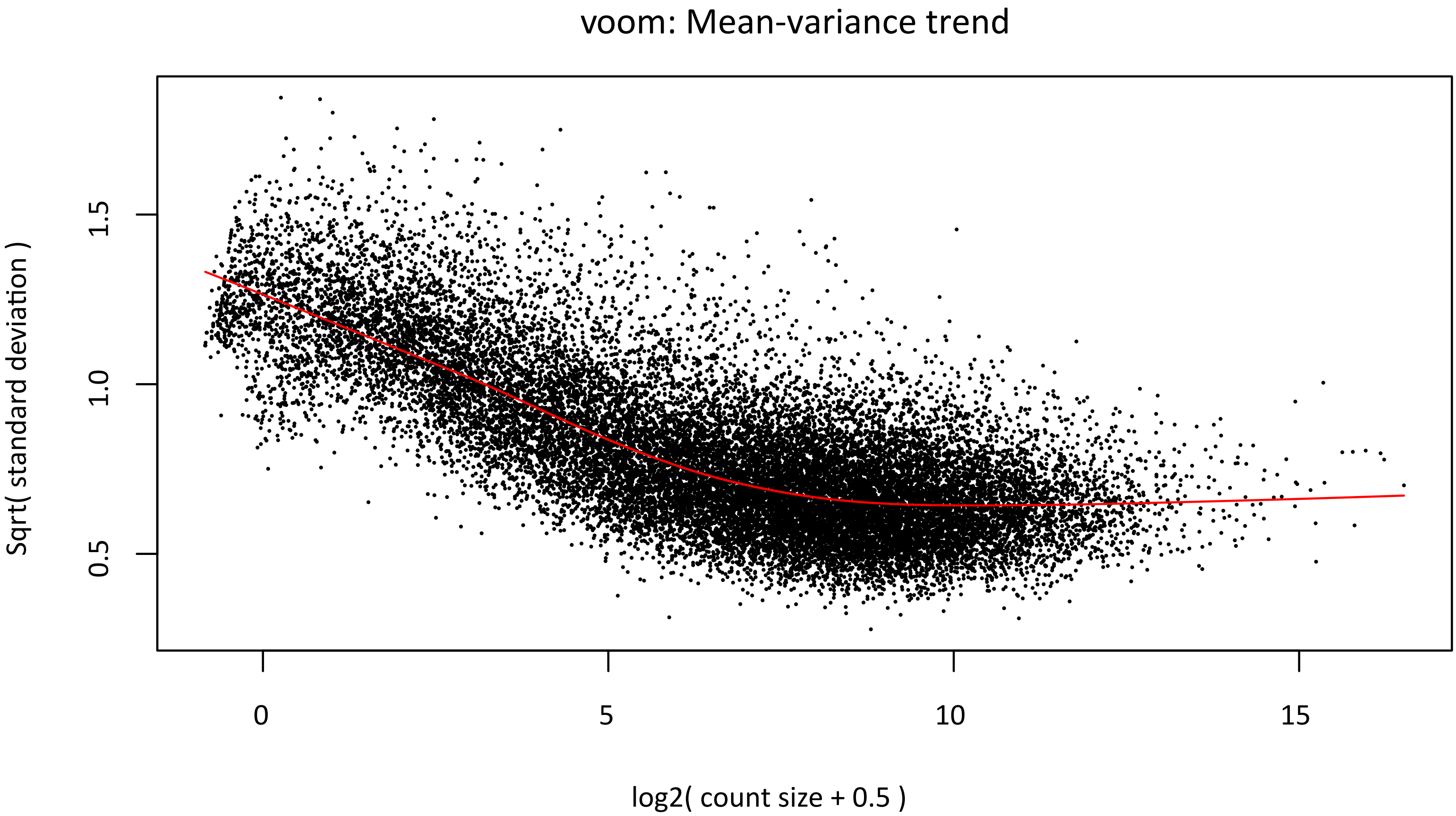

Supplemental Figure S2. Scatterplots of the distribution of means (x-axis) and variances (y-axis) of each gene showing the dependence between the two before (A) and after (B) Voom is applied to the data.

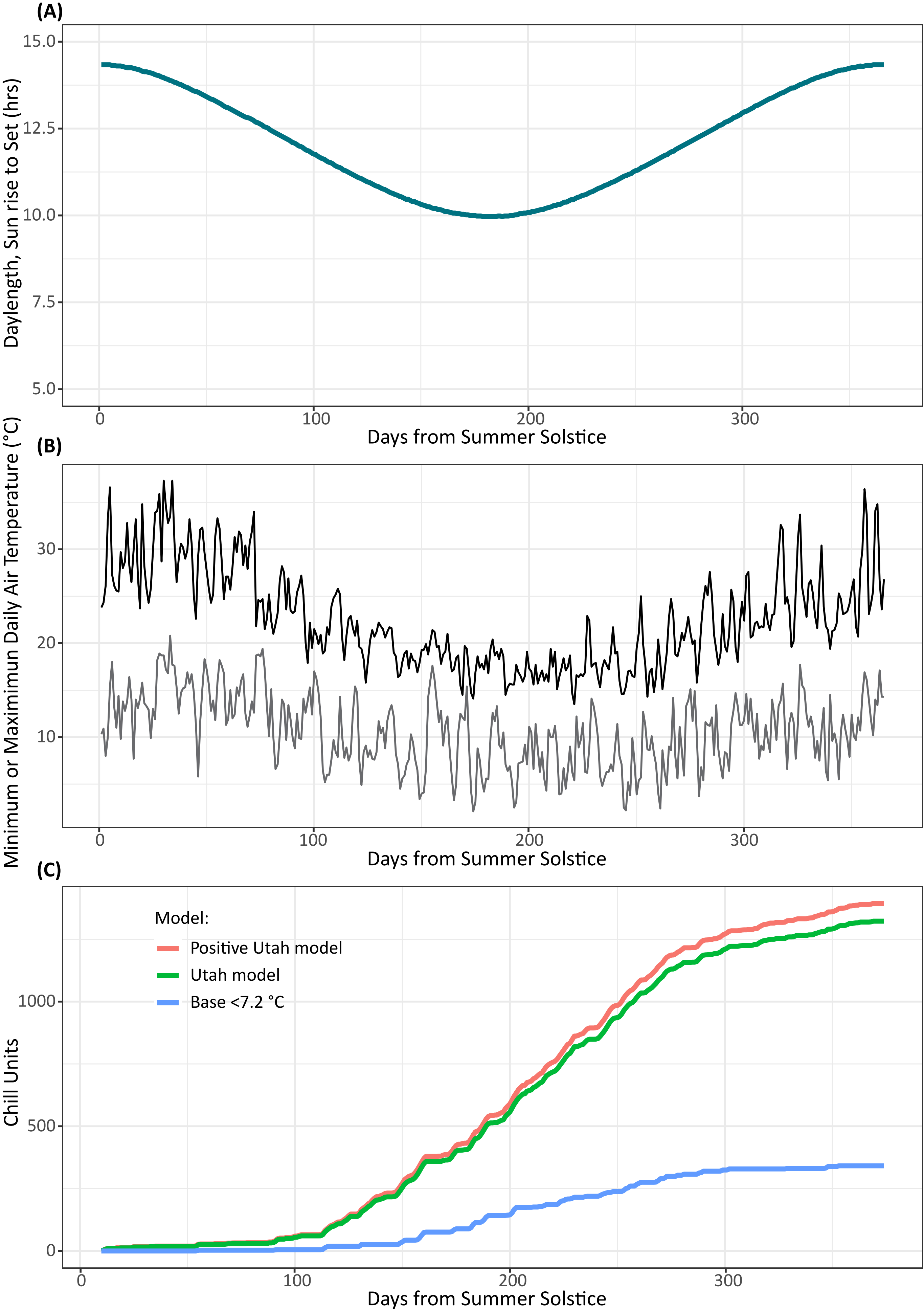

Supplemental Figure S3. Seasonal changes in photoperiod (A), temperature (B) and cumulative chilling (C) in the Margaret River region of Western Australia (2015).
